# Epigenetic Impacts of Ascorbate on Human Metastatic Melanoma Cells

**DOI:** 10.3389/fonc.2014.00227

**Published:** 2014-08-25

**Authors:** Sascha Venturelli, Tobias W. Sinnberg, Alexander Berger, Seema Noor, Mitchell Paul Levesque, Alexander Böcker, Heike Niessner, Ulrich M. Lauer, Michael Bitzer, Claus Garbe, Christian Busch

**Affiliations:** ^1^Department of Internal Medicine I, Medical University Hospital, Tuebingen, Germany; ^2^Division of Dermatologic Oncology, Department of Dermatology and Allergology, University of Tuebingen, Tuebingen, Germany; ^3^Department of Dermatology, University Hospital Zurich, Zurich, Switzerland; ^4^Evotec AG, Hamburg, Germany

**Keywords:** ascorbate, vitamin C, cancer, melanoma, epigenetics, microRNA, HDAC, DNMT

## Abstract

In recent years, increasing evidence has emerged demonstrating that high-dose ascorbate bears cytotoxic effects on cancer cells *in vitro* and *in vivo*, making ascorbate a pro-oxidative drug that catalyzes hydrogen peroxide production in tissues instead of acting as a radical scavenger. This anticancer effect of ascorbate is hypoxia-inducible factor-1α- and O_2_-dependent. However, whether the intracellular mechanisms governing this effect are modulated by epigenetic phenomena remains unknown. We treated human melanoma cells with physiological (200 μM) or pharmacological (8 mM) ascorbate for 1 h to record the impact on DNA methyltransferase (DNMT)-activity, histone deacetylases (HDACs), and microRNA (miRNA) expression after 12 h. The results were analyzed with the MIRUMIR online tool that estimates the power of miRNA to serve as potential biomarkers to predict survival of cancer patients. FACS cell-cycle analyses showed that 8 mM ascorbate shifted BLM melanoma cells toward the sub-G1 fraction starting at 12 h after an initial primary G2/M arrest, indicative for secondary apoptosis induction. In pharmacological doses, ascorbate inhibited the DNMT activity in nuclear extracts of MeWo and BLM melanoma cells, but did not inhibit human HDAC enzymes of classes I, II, and IV. The expression of 151 miRNAs was altered 12 h after ascorbate treatment of BLM cells in physiological or pharmacological doses. Pharmacological doses up-regulated 32 miRNAs (≥4-fold) mainly involved in tumor suppression and drug resistance in our preliminary miRNA screening array. The most prominently up-regulated miRNAs correlated with a significantly increased overall survival of breast cancer or nasopharyngeal carcinoma patients of the MIRUMIR database with high expression of the respective miRNA. Our results suggest a possible epigenetic signature of pharmacological doses of ascorbate in human melanoma cells and support further pre-clinical and possibly even clinical evaluation of ascorbate for melanoma therapy.

## Introduction

In recent years, a large number of studies demonstrated that in pharmacological doses, ascorbic acid (ascorbate, vitamin C) in the low micromolar-range exerts cytotoxic effects on cancer cells *in vitro* and *in vivo* (1–3) via pro-oxidative mechanisms (4). This cytotoxicity is conducted by ascorbyl radicals and H_2_O_2_ being catalyzed by serum components (5). Hypoxic conditions and hypoxia-inducible factor-1α (HIF-1α)-signaling, both present in cancer metastases, confer resistance to the cancer cells toward ascorbate-induced cytotoxicity (5), while ascorbate inhibits HIF-1 with mechanisms of iron competition (6). This bears a strong clinical implication, since increased tumor ascorbate is associated with extended disease-free survival and decreased HIF-1 activation in human colorectal cancer (7). Likewise, low ascorbate levels are associated with increased HIF-1 activity and an aggressive tumor phenotype in endometrial cancer (8). Interestingly, ascorbate has a preferential toxicity toward melanoma cells (9). In B16, melanoma-bearing mice spontaneous lung metastasis is inhibited by sodium ascorbate supplementation in drinking water in mice fed a restricted diet (low in tyrosine and phenylalanine) (10). *In vitro*, the induction of a pro-oxidant state by ascorbate and a subsequent reduction in mitochondrial membrane potential are involved in a caspase-8-independent apoptotic pathway of B16F10 melanoma cells (11). Further, oral ascorbate supplementation modulates B16FO melanoma growth, metastasis, and inflammatory cytokine secretion as well as enhanced encapsulation of tumors in scorbutic (l-gulono-gamma lactone oxidase −/−) mice (12, 13).

In this respect, we recently demonstrated that patients afflicted with metastatic melanoma (stage IV) have lower plasma ascorbate levels compared to healthy controls and that polychemotherapy or immunotherapy further decreases plasma ascorbate levels in stage IV melanoma patients (14). However, the ascorbate concentration required for cytotoxicity in cancer cells can only be achieved via intravenous (i.v.) administration (15); up to 49 mM ascorbate blood peak concentrations are thus achievable by administration of 70 g/m^2^ (16). Yet, in recent phase I clinical trials, ascorbate failed to demonstrate a significant anticancer activity (16–19), although it enhanced chemosensitivity of ovarian cancer cells and reduced toxicity of chemotherapy (20). This obvious discrepancy between impressive anticancer efficacy in various pre-clinical models and lack of a reproducible anticancer activity in cancer patients clearly demonstrates that crucial (co-)factors executing the anticancer efficacy and an appropriate clinical treatment regimen remain to be deciphered. Due to the broad concentration range of ascorbate in humans and its numerous biochemical functions and effects, which seem to differ in somatic and malignantly transformed human cells (1), further research is needed for the understanding of the precise cytotoxic molecular impacts of ascorbate in cancer cells.

Many naturally occurring compounds and nutrients exert beneficial anticancer effects (e.g., suppression of tumor growth or induction of apoptosis), some of which are linked to modulation of epigenetic mechanisms (21–23). In general, epigenetic modifications influence gene expression without altering the DNA sequence and are therefore potentially reversible. Several epigenetic changes were distinguished, including histone acetylation and DNA methylation, and are currently investigated as potential targets for anticancer therapy (24). Both of the latter regulate the expression of microRNAs (miRNAs) and at the same time, are in part controlled by miRNAs via a regulatory circuit (25, 26). miRNAs correlate with clinical outcome in cancer patients in clinical studies (27). To test the possible relation between the expression of any given miRNA and the clinical outcome of cancer patients, the free online MIRUMIR tool, which performs survival analyses and draws Kaplan–Meier plots for any miRNA across several available data sets, was recently established (28).

In the present study, we provide novel evidence that in human metastatic melanoma cells only pharmacological doses of ascorbate induce substantial epigenetic changes. For 8 mM ascorbate, we detected a moderate inhibition of cellular histone deacetylase (HDAC) enzymes and a prominent DNA methyltransferase (DNMT) inhibition. Only pharmacological doses of ascorbate seemed to alter the miRNA expression profile by up-regulating 32 miRNAs mainly involved in tumor suppression and drug resistance, as demonstrated by preliminary miRNA chip expression analyses. Together, our results suggest that high doses of ascorbate only achievable in patients by i.v. administration might have epigenetic impacts on melanoma cells that might be beneficial in combination with classical or novel therapeutic anticancer approaches.

## Materials and Methods

### Cell lines and chemicals

Metastatic melanoma cell lines [MeWo: derived from a lymph node metastasis of a 78 years Caucasian donor; mutation status: BRAF wild-type, NRAS wild-type (29), BLM: subline of BRO melanoma cells isolated from lung metastases after subcutaneous inoculation of nude mice with BRO cells; mutation status: BRAF wild-type, NRAS mutated (30)] were cultured in RPMI 1640 medium supplemented with 10% fetal bovine serum (FBS), 1% penicillin and streptomycin, and 1% l-glutamine. All cell culture experiments were performed at 37°C and 5% CO_2_. The following chemical was used: injectable vitamin C solution (Pascorbin^®^, 150 mg ascorbate/1 ml injection solution, pH 7.0; Pascoe pharmazeutische Praeparate GmbH, Giessen, Germany).

### Cell-cycle analysis

BLM cells were incubated with ascorbate at 8 mM. After 0–24 h (in 2 h intervals) the cells (1 × 10^6^) were harvested, washed with cold PBS, fixed with 75% ethanol, and incubated at 4°C for at least 1 h. Cells were then centrifuged and washed twice in cold PBS. Intracellular DNA was labeled with propidium iodide solution [propidium iodide 40 mg/ml (Sigma) and RNase 100 mg/ml (Thermo Scientific) in PBS] and incubated at 4°C for 30 min in the dark. Cell cycle was analyzed using flow cytometry and FACSDiva software (BD Biosciences, Heidelberg, Germany).

### *In silico* (docking-) analysis of histone deacetylase inhibition

#### Ligand preparation

**Figure 1:**
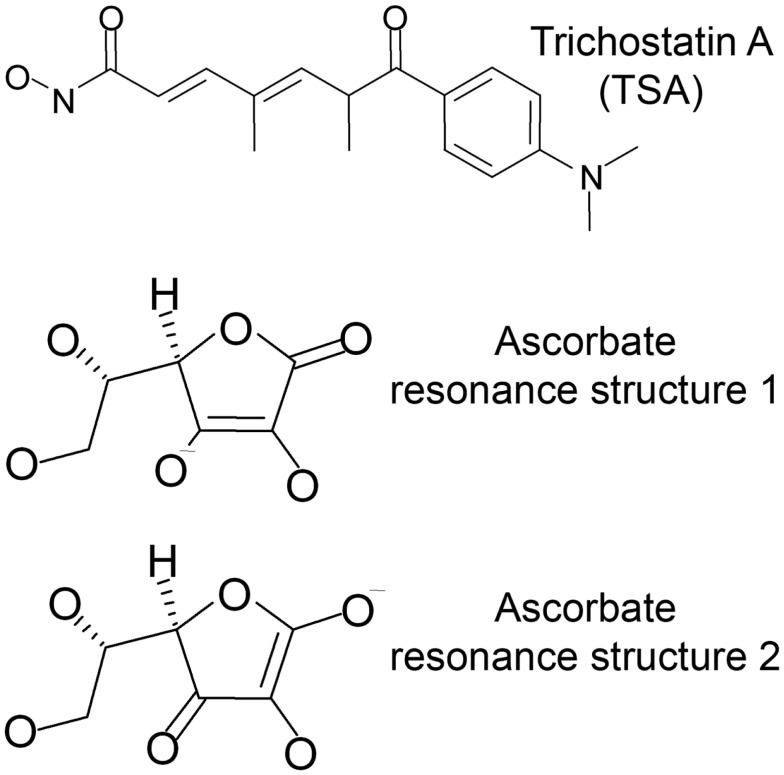
**Ligands used for docking into the crystal structures of HDAC-2, -4, -7, and -8**.

For this study, docking was performed into human HDACs 2, 4, 7, and 8 with trichostatin A (TSA) and the two major resonance structures of ascorbic acid (Figure 1). All ligands were prepared using the molecular operation environment (MOE, version 2007.09, Chemical Computing Group, Inc., Montreal, QC, Canada). 3D representations of the ligands were obtained by energy minimization (Rebuild3D function with preservation of existing chiral centers) using MM94x force field and a Born Solvation model without cutoff constraints. All other parameters were left at default.

#### Protein preparation

Crystal structures of HDAC2 (PDB code: 3max), HDAC4 (PDB code: 2vqm), HDAC7 (PDB code: 3c10), and HDAC8 (PDB code: 1t64) were retrieved from the protein data bank^1^ (PDB) and loaded into MOE. The Protonate3D functionality was applied to assign the correct ionization state and geometries to the protein atoms and to add hydrogen atoms (31). For the final docking, water molecules were discarded.

#### Docking

Docking was performed using GOLD (version 4.1.2, The Cambridge Crystallographic Data Centre, Cambridge, UK). No additional protein preparation was applied. Binding sites were defined by all residues within 5 Å distance from the corresponding ligands in the crystal structure. Docking was performed using GoldScore as scoring function. All other parameters were left at default. Docking poses were analyzed in MOE. To optimize the ligand–receptor interactions energy minimizations were applied using MM94x force field and a Born Solvation model without cutoff constraints.

### HDAC-inhibitor screening assay

Determination of a possible HDAC-inhibitor activity of ascorbate was done by using the HDAC assay kit (Active Motif, Rixensart, Belgium). Ascorbate was diluted in assay buffer to the final concentrations of 5, 10, 20, 50, 100, 200 μM, and 8 mM. Assay was performed according to manufacturer’s protocol. Briefly, ascorbate was incubated with HeLa nuclear extract as a source of human HDACs for 2 h at 37°C and the developing time was set to 10 min. Each experiment was performed in triplicates and repeated three times.

### HDAC-inhibitor profiling assay

The HDAC profiling assay was performed on basis of fluorometric measurement by Scottish Biomedical (Scottish Biomedical, Glasgow, UK). The percentage inhibition values of 50 μM and 8 mM ascorbate against human HDAC1, HDAC2, HDAC3, HDAC4, HDAC5, HDAC6, HDAC7, HDAC8, HDAC9, HDAC10, and HDAC11 was determined. Both concentrations of ascorbate were tested in two experiments, each in duplicates. TSA is used as a standard inhibitor by Scottish Biomedical for this assay and was deployed according to the information of the manufacturer in the following concentrations; HDAC1, HDAC2, HDAC3, HDAC6, HDAC10, and HDAC11 were tested at 10 nM TSA, HDAC8 at 100 nM and HDAC4, HDAC5, HDAC7, and HDAC9 were tested at 10 μM TSA.

### Measurement of DNA methyltransferase activity

Nuclear extracts were prepared from BLM and MeWo melanoma cells (in triplicates) 12 h after 1 h treatment with ascorbate (untreated, 200 μM and 8 mM) by using the Nuclear Extract Kit (Active Motif) according to the procedure described by the manufacturer. DNMT activity was analyzed in the nuclear extracts with the DNMT activity/inhibition assay (Active Motif) according to the procedure described by the manufacturer.

### miRNA expression analysis

microRNA was isolated from BLM cells using the miRNeasy kit (Qiagen, Hilden, Germany) according to the procedure described by the manufacturer. miRNA expression analysis was performed on BLM melanoma cells (five groups: untreated; 200 μM, 8 mM ascorbate treated, 4 and 12 h after the 1 h treatment, all in triplicates) using the human miRNA Microarray Release 14.0, 8x15K (Agilent, Waldbronn, Germany) based on Sanger miRbase (release 14.0). Two hundred nanograms of RNA were used per sample. The miRNA expression analysis was kindly performed at the Genomic Core Facility of the European Molecular Biology Laboratory (EMBL, Heidelberg, Germany) according to the supplier’s instructions. Evaluation of raw data generated at the Genomic Core Facility of the EMBL was performed as described previously (32).

### MIRUMIR miRNA analysis

Five highly up-regulated miRNA 12 h after 8 mM ascorbate treatment (miR-596, miR-630, miR-490, miR-375, and miR-708) were analyzed using the free online MIRUMIR database (28), which is incorporated into BioProfiling.de, an analytical portal for high-throughput cell biology^2^. The MIRUMIR database draws Kaplan–Meier plots for the submitted miRNAs after an implemented statistical procedure to account for multiple testing; *P*-values are generated automatically.

### Statistical analysis

Statistical analysis was performed with One-way ANOVA Dunnett’s multiple comparison test using GraphPad Prism version 4.00 (GraphPad Software, San Diego, CA, USA). According to One-way ANOVA Dunnett’s multiple comparison test, all ascorbate treatment groups were compared vs. vehicle/control. All values of *P* > 0.05 were defined as statistically not significant. The miRNA chip array was analyzed as described previously in detail (32).

## Results

### Pharmacological ascorbate induces apoptosis in human metastatic BLM melanoma cells in a time-dependent manner

**Figure 2:**
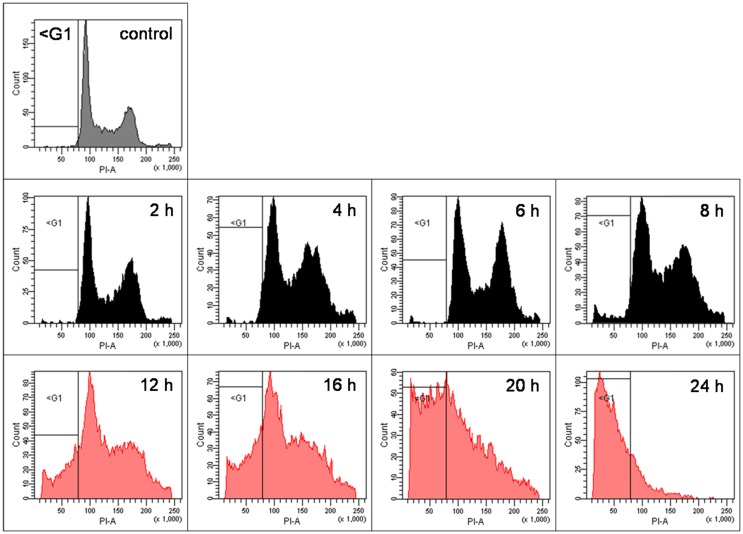
**Pharmacological ascorbate induces apoptosis in human metastatic BLM melanoma cells in a time-dependent manner**. BLM cells were treated with 8 mM ascorbate for 1 h. Cells were ethanol-fixed every 2 h for 24 h, stained with propidium iodide, and cell cycle was analyzed with FACS. Depicted are the FACS plots for the untreated control cells and for 2, 4, 6, 8, 12, 16, 20, and 24 h after ascorbate exposure. The G2/M fraction of cells steadily increased from 2 to 8 h, while 12 h post-treatment a prominent increase of the sub-G1 fraction of DNA fragmented cells started (indicative for apoptosis induction). At 20 h post-treatment, the cell cycle was already completely shifted toward the sub-G1 fraction. Gray colored graphs indicate the control cells; black colored graphs (2–8 h) the initial shift into G2/M phase; red colored graphs (12–24 h) the subsequent shift into sub-G1 fraction.

As shown previously (5), pharmacological doses of ascorbate in the low millimolar-range induce cell death in human cancer cells. However, since in the latter publication we only performed end-point analyses 24 h after incubation of the cells with ascorbate, for the current project we exposed BLM cells to pharmacological 8 mM ascorbate for 1 h, and the cells were ethanol-fixed every 2 h for 24 h to closely monitor cell-cycle alterations at 2 h intervals over 24 h. The cell cycle was analyzed with FACS after staining of the cells with propidium iodide. We observed that the G2/M fraction of cells initially steadily increased starting at 2 h after ascorbate exposure, while 12 h post-treatment a subsequent increase of the sub-G1 fraction of DNA fragmented cells was evident (indicative for apoptotic cells). At 20 h post-treatment, the cell cycle was already completely shifted toward the sub-G1 fraction (Figure 2). At 12 h post ascorbate exposure only a small percentage of the BLM cells were shifted toward the sub-G1 fraction of cells. Therefore, the 12 h time point after ascorbate exposure was chosen for the following experiments.

### Pharmacological ascorbate moderately inhibits histone deacetylases

Epigenetic modifications such as histone acetylation or DNA methylation play an important role in cancer development and progression (24). Whether ascorbate has epigenetic effects on cancer cells has not been investigated yet. However, since reactive oxygen species (ROS) induce hypermethylation of the E-cadherin promoter regions in hepatoma cells (33), we hypothesized that ascorbate, a pro-oxidative radical-inducing drug in pharmacological concentrations (2, 5), might bear similar epigenetic effects on melanoma cells. To verify our hypothesis, we therefore analyzed two major epigenetic mechanisms, inhibition of DNMTs and HDACs.

**Figure 3:**
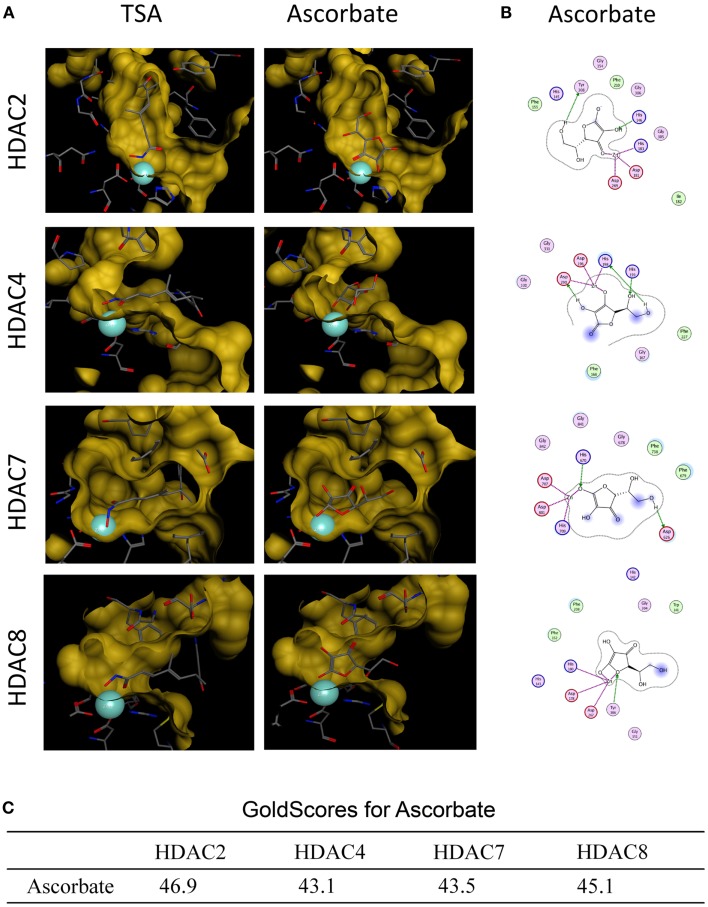
***In silico* docking analysis of ascorbate and HDACs**. **(A)** *In silico* docking analyses of ascorbate and HDAC2, HDAC4, HDAC7, and HDAC8. Trichostatin A (TSA) served as positive control. The analysis demonstrates the fitting of ascorbate into each HDAC binding pocket and the ability to interact with the HDAC-derived zinc ion (turquoise sphere) of the catalytic center. **(B)** 2D depiction of ligand is shown along with interacting amino acids. Green circles represent greasy, purple circles polar, red circles acidic, and blue circles basic amino acids. HDAC contacts are depicted by a blue half moon around the amino acids. Blue arrows represent backbone acceptors, green ones depict side chain acceptors and side chain donors. Green benzoyl rings with a “+” describe an arene–cation binding, two benzoyl rings an arene–arene binding. Areas with a blue background are exposed to the ligand. The purple dotted lines represent metal contact. **(C)** Docking analysis of ascorbate in the individual HDAC binding pockets were performed using GOLD software (version 4.1.2) and MOE.

**Figure 4:**
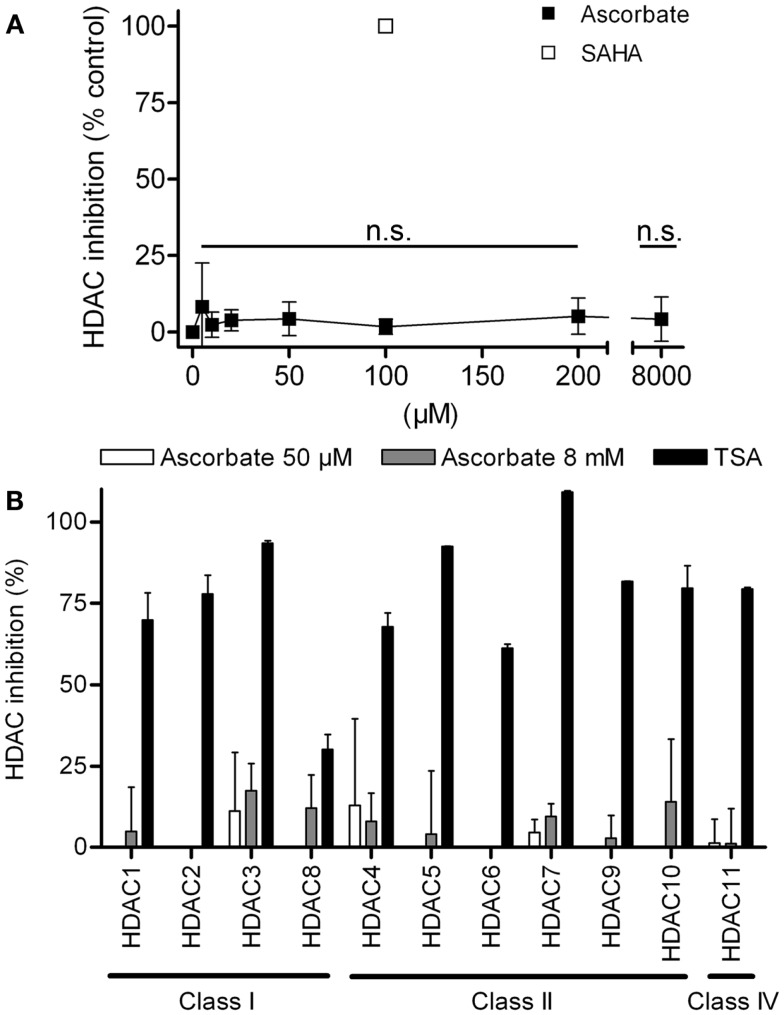
**Ascorbate does not act as histone deacytelase inhibitor (HDACi)**. **(A)** Overall HDAC inhibition in cellular extracts of the human cell line HeLa by increasing concentrations of ascorbate (5–8000 μM). As reference inhibitor 100 μM suberoylanilide hydroxamic acid (SAHA) was used. Every concentration was tested three times in triplicates. **(B)** Specific fluorometric profiling assay using recombinant human HDACs of classes I, II, and IV. Specific inhibition values were generated for the treatment with 50 μM and 8 mM ascorbate. Inhibition values for every HDAC were yielded by two experiments, each performed in duplicates. Shown are mean ± SD. One-way ANOVA Dunnett’s multiple comparison test, n.s. indicates not significant.

First we analyzed, if ascorbate possessed an HDAC-inhibitory activity. To this end, an *in silico* docking analysis was performed. The *in silico* analysis revealed that ascorbate was able to penetrate into the binding pocket of class I and II HDACs and to interact with the zinc ion, two issues that are important for HDAC inhibitors (Figures 3A,B). Calculated GoldScores representing the binding affinity, supported these first data, leading us to the assumption that ascorbate, in a given setup, could be a similar strong binding partner to the binding pockets as the well-known HDAC-inhibitor TSA (Figure 3C) (22). Due to the positive results obtained by the docking experiments, we next performed a cell free HDAC-inhibitor assay. In this assay, nuclear extract of the well-characterized human HeLa cell line was used as HDAC enzyme source. The results showed that in contrast to the *in silico* docking data, *in vitro* only a marginal inhibitory activity of ascorbate on HDACs could be detected (Figure 4A). The latter data could be verified by a profiling of all known HDAC enzymes of class I, II, and IV. As before, TSA was used as reference HDAC inhibitor in this experimental setting. In line with the HDAC-inhibitor assay above, neither the physiological 50 μM nor the pharmacologic 8 mM ascorbate showed a significant inhibition of the 11 conserved human HDACs tested when compared to the potent inhibition mediated by TSA (Figure 4B).

### Pharmacological ascorbate inhibits DNA methyltransferases

**Figure 5:**
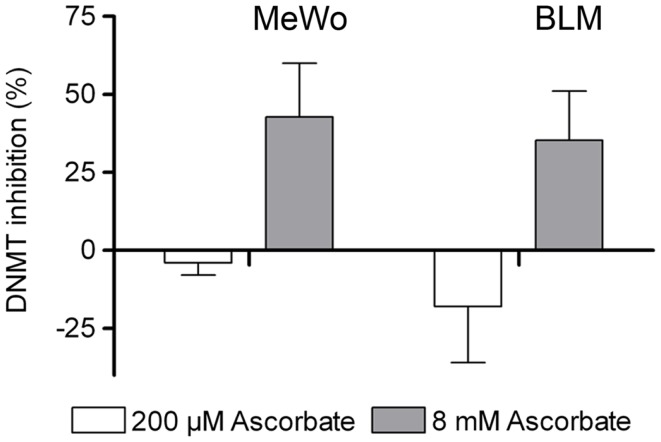
**Ascorbate inhibits DNA methyltransferase (DNMT) activity in melanoma cells**. Global DNMT activity was determined in nuclear extracts of MeWo and BLM cells 12 h after 1 h treatment with 200 μM or 8 mM ascorbate. Treatment with 200 μM ascorbate increases DNMT activity in MeWo and BLM cells by 4 and 18%, respectively, and 8 mM ascorbate inhibits DNMT activity by 43 and 35%, respectively.

Due to the negative results of the HDAC inhibition assays, we next investigated if ascorbate had a DNMT inhibitory activity in the human metastatic MeWo and BLM melanoma cells. Twelve hours after treatment of the respective melanoma cells with either physiological 200 μM or pharmacological 8 mM ascorbate for 1 h, a nuclear extract was prepared and the amount of methylated DNA was measured. The experiments showed that the physiological concentration of ascorbate (200 μM) increased the DNMT activity in a moderate fashion in both cell lines. Interestingly, the pharmacological concentration of 8 mM ascorbate clearly inhibited DNMTs in both cell lines by up to 40% (Figure 5).

### Pharmacological ascorbate strongly modifies miRNA expression

**Figure 6:**
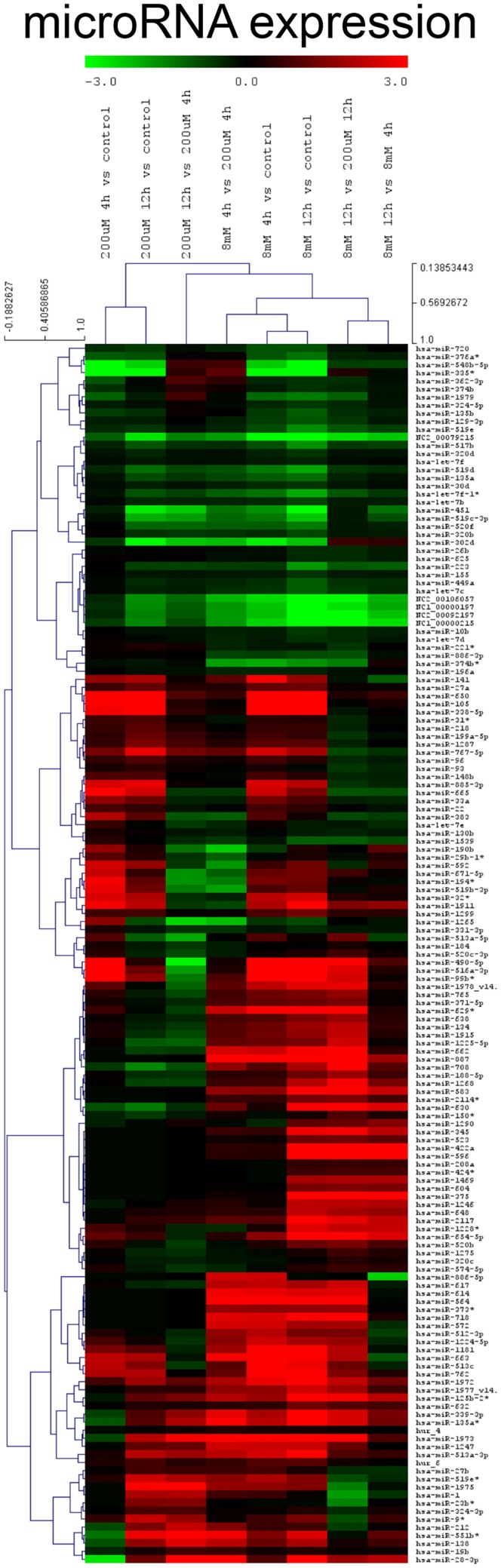
**Ascorbate alters the expression of miRNA in melanoma cells**. A miRNA expression chip analysis was performed on human metastatic BLM melanoma cells (5 groups: untreated, 200 μM ascorbate, 8 mM ascorbate, both 4 and 12 h after ascorbate exposure; all in triplicates) using the human miRNA Microarray Release 14.0, 8x15K (Agilent Technologies) based on Sanger miRbase (release 14.0). A total of 151 miRNAs were differentially expressed in response to ascorbate. Incubation of BLM cells with 8 mM ascorbate for 1 h up-regulated 32 miRNAs (4- to 38-fold) involved in tumor suppression and drug resistance compared to physiological (200 μM) ascorbate after 12 h.

**Figure 7:**
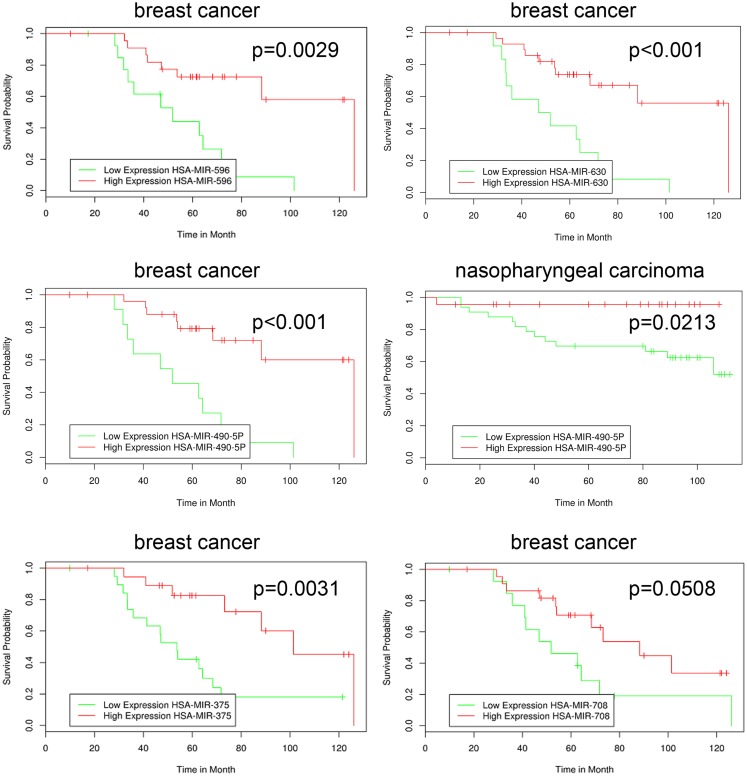
**High expression of miRNA up-regulated by ascorbate in melanoma cells correlates with increased overall survival in cancer patients**. The MIRUMIR online database was screened to detect an impact of the up-regulated miRNA (compare Figure 6) on cancer patient survival. The depicted Kaplan–Meier plots are automatically drawn by MIRUMIR upon submission of the respective miRNAs. A significant correlation of high expression of miR-596, miR-630, miR-490, miR-375, and miR-708 with overall long-term survival in breast cancer (GEO dataset IDs: GSE37405 and GSE37405) or nasopharyngeal carcinoma patients (GEO dataset ID: GSE36682) was observed when compared to low expression of the respective miRNA.

The novel classification of pharmacological ascorbate as DNMT inhibitor rose the question if the inhibition of DNMTs within the melanoma cells subsequently had an impact on the cellular miRNA expression profile. To answer this question, we performed miRNA expression chip analysis. The investigation revealed that the expression of 151 miRNAs was significantly altered when comparing ascorbate treatment for 1 h at 8 mM with treatment at 200 μM (Figure 6). The IC_50_ of ascorbate is in the low millimolar range in melanoma cells (5). Since in this study, we mainly investigated the role of epigenetic mechanisms accompanying ascorbate-induced cytotoxicity, we did not focus on miRNA expression changes induced by physiological ascorbate (200 μM), which bears no cytotoxic effect on melanoma cells. Comparing the impact of the pharmacological dose of ascorbate (8 mM) with the impact of ascorbate at the maximum physiological plasma condition of 200 μM, after 12 h a significant up-regulation of 32 miRNAs (4- to 38-fold) could be stated. Interestingly, 14 of these miRNAs (miR-596, miR-630, miR-422a, miR-490-5p, miR-375, miR-708, miR-345, miR-125b-2, miR-516a-3p, miR-135a, miR-1228, miR-1915, miR-134, and miR-663) have established roles in tumor suppression and drug resistance, while 5 miRNAs (miR-630, miR-375, miR-345, miR-1228, and miR-134) are known to inhibit epithelial–mesenchymal transition and invasion in cancer cells. Eleven of the up-regulated miRNAs (miR-887, miR-583, miR-662, miR-1973, miR-718, miR-1268, miR-2117, miR-614, miR-617, miR-1972, and miR-1181) have no reported functions in cancer cells yet. A detailed list of the 32 up-regulated miRNAs, their reported expression profile in cancer, functions and predicted RNA-targets are given in Table 1. To further analyze a possible clinical significance of the up-regulated miRNA upon ascorbate administration, we screened the free MIRUMIR online database (28), which tests any given miRNA as biomarker to predict survival in available clinical data sets that cover more than 800 cancer patients. We were able to find a strong correlation of high expression of miR-596, miR-630, miR-490, miR-375, and miR-708 with overall long-term survival in breast cancer or nasopharyngeal carcinoma patients when compared to low expression of the respective miRNA in the same cohorts of patients. The Kaplan–Meier plots as depicted in Figure 7 were automatically generated by MIUMIR upon submission of the respective miRNAs (miR-596, miR-630, miR-490, miR-375, and miR-708).

**Table 1:** **miRNA expression profile of BLM melanoma cells after ascorbate treatment**.

miRNA	Up-regulation (2 × log^2^)	Expression in cancer	Function	Predicted targets (mir SVR score^a^)	Reference
hsa-miR-596	5.26	Urothelial carcinoma 174 UC and 33 UC cells Ependymoma Hepatocellular carcinoma tissue Oral squamous cell carcinoma	Candidate tumor suppressor gene region Expression correlates with survival Expression correlates with survival Tumor suppressor *in vivo*	ABCB5 (multidrug resistance exporter, over-expressed in melanoma)	(34) (35) (36) (37)
hsa-miR-887	5.14	N/A		PDK1 (Akt pathway), FN1 (c-MET/HGF-pathway), MAP3K1 (apoptosis)	
hsa-miR-630	4.3	Non-small cell lung cancer A549 cells	Modulates mitochondrial/post-mitochondrial steps of the intrinsic pathway of apoptosis; blocks early manifestations of the DNA damage response	IGF2BP3 (proliferation), CDK1 (interacts with FOXO1a, tumor suppression), FANCI (DNA repair), EP300 (MITF-pathway), Wnt/b-catenin, SLUG	(38)
		Lung cancer	Suppresses SLUG *in vivo* and thus epithelial mesenchymal transition in an integrin α(1)β(1)/FAK/ERK/SP1 pathway-dependent manner		(39)
		Pancreas cancer cells	Induces apoptosis in pancreatic cancer cells by targeting IGF-1R		(40)
hsa-miR-422a	4.29	Osteosarcoma tissue and cells	Up-regulation predicts tumor sensitivity to ifosfamide	RBX1 (proteasomal degradation)	(41)
hsa-miR-583	3.97	N/A		KIT, RCC1 (oncogenes)	
hsa-miR-490-5p	3.91	Bladder cancer tissue	Down-regulated in bladder cancer	PI3K (mTOR/AKT pathway), NGR1 (invasiveness), IL7 (activates JAK/STAT5), PTPRD (tumor suppression)	(42)
hsa-miR-375	3.65	Pancreatic ductal adeno-carcinoma tissue and cells	Down-regulated in pancreas cancer		(43)
		Gastric cancer tissue and cells	Tumor suppressor regulating gastric cancer cell proliferation		(44, 45)
		Hepatocellular carcinoma tissue	Inhibits proliferation and invasion of HCC cells via suppression of endogenous YAP oncogene protein level		(46)
		Head and neck squamous cell carcinoma tissue and cells	Down-regulated in head and neck squamous cell cancer		(47)
		Esophagus squamous cell and adeno-carcinoma tissues	Down-regulation is associated with worse prognosis		(48)
		Cervical cancer cell lines	Tumor suppressor in cervical carcinogenesis		(49, 50)
hsa-miR-662	3.59	N/A			
hsa-miR-708	3.48	Colon carcinoma tissue and cells	Expressed in colon carcinoma, regulates oncogenetic (MAPK, PI3K) pathways	IKBKB (NFκB activation), SPARC (invasiveness, EMT induction), ANXA1 (migration)	(51)
		Renal cell carcinoma	Tumor suppressor in renal cell carcinoma *in vivo*		(52)
		Prostate cancer	Decreases tumorigenicity of CD44(+) prostate cancer-initiating cells *in vitro* and *in vivo*		(53)
		Glioblastoma	Tumor suppressor in human glioblastoma cells		(54)
hsa-miR-654-5p	3.39	Prostate cancer cells	Regulates expression of androgen receptor	AKT (proliferation), notch-1 (oncogene in melanoma)	(55)
hsa-miR-629	3.38	Breast, colon, liver, lung, lymphoma, ovary, prostate, testis cancer tissue	Up-regulated in various cancers	ZBTB16 (melanoma progression), PPARG (apoptosis induction)	(56, 57)
hsa-miR-564	3.28	Chronic myeloid leukemia cells	Down-regulated in chronic myeloid leukemia cells		(58)
hsa-miR-1973	3.08	N/A		SHC4 (RAS activation)	
hsa-miR-718	2.97	N/A			
hsa-miR-1268	2.84	N/A			
hsa-miR-345	2.82	Breast adeno-carcinoma MCF-7 cells	Targets the human multidrug resistance-associated protein 1		(59)
		Colon cancer cells	Suppresses colon cancer cell proliferation and invasiveness	BCL2-associated athanogene 3 (BAG3)	(60)
hsa-miR-125b-2	2.8	Large cell lung carcinoma Calu-6 cells	Putative tumor suppressor residing in the commonly deleted 21q21 region	TNF (proinflammatory cytokine)	(61)
hsa-miR-2117	2.65	N/A		SPP1 (invasiveness, EMT, over-expressed in melanoma)	
hsa-miR-614	2.61	N/A			
hsa-miR-516a-3p	2.56	Ovarian cancer cells	Decreases cell proliferation via decrease of kallikrein-related peptidases (KLKs)	ABCB5 (multidrug resistance exporter, over-expressed in melanoma)	(62)
hsa-miR-339-3p	2.47	B-cell precursor acute lymphoblastic leukemia cells	Over-expressed in pre-B-ALL patients		(63)
hsa-miR-135a	2.45	Non-small lung carcinoma cells	Involved in paclitaxel resistance	MS4A1 (B-cell activation), MCL1	(64)
			Sensitizes A549 lung cancer cells for cisplatin-induced apoptosis		(65)
hsa-miR-99b	2.44	Primary melanoma tissues	Increased expression in melanomas of older patients		(66)
		Esophageal cancer	Up-regulated in esophageal cancer		(67)
hsa-miR-1225-5p	2.38	Prostate cancer cells	Androgen-regulated in prostate cancer cells		(68)
hsa-miR-617	2.33	N/A			
hsa-miR-1228	2.24	Malignant mesothelioma tissue	Up-regulated in malignant mesothelioma	CK2A2	(69)
		Gastric cancer	Suppressed gastric cancer formation *in vivo*, suppresses epithelial mesenchymal transition		(70)
hsa-miR-1915	2.22	Human embryonal stem cells	Inhibits notch-1 *in silico*	BCL2	(71)
		Colon carcinoma cells	Sensitizes HCT116 colon cancer cells to anticancer drugs		(72)
hsa-miR-1972	2.22	N/A			
hsa-miR-134	2.15	Small cell lung cancer NCI-H69 and NCI-H69AR cells	Reduces sensitivity to cisplatin, etoposide and doxorubicin by induction of G1 arrest	FOXM1, Nanog, KRAS	(73)
		Non-small cell lung cancer cells	Inhibits epithelial mesenchymal transition		(74)
		Glioblastoma	Down-regulated in glioblastoma		(75)
		Hepatocellular carcinoma	Suppresses HCC *in vivo* by down-regulation of KRAS		(76)
hsa-miR-1246	2.13	Malignant mammary epithelial cells	Released into blood, milk, and ductal fluids, possible biomarker		(77)
hsa-miR-1181	2.06	N/A			
hsa-miR-663	2.05	Colon cancer SW480 cells	Resveratrol-induced tumor suppressor targeting TGFb1 transcripts		(78)
		Gastric cancer BGC823 and SNU5 cells	Tumor suppressor in gastric cancer cells		(79)
		Melanoma tissue samples	Up-regulated in melanoma		(80)

^a^http://www.microrna.org/microrna/home.do

*List of differentially expressed miRNAs 12 h after exposure of BLM cells to 8 mM ascorbate for 1 vs. 12 h after exposure to 200 μM ascorbate for 1 h, up-regulation >2 × log^2^*.

## Discussion

Cutaneous melanoma is an aggressive malignancy with increasing incidence. Up to now curative therapies for stage IV patients, which have an overall survival of 9–14 months, are lacking (81–83). Therefore, in spite of the recently approved targeted drugs (BRAF- or MEK-inhibitors) or available immunotherapies (anti-CTLA-4- or anti-PD1-antibodies) novel therapeutic strategies are still urgently needed. Numerous alternative treatment approaches are therefore currently investigated in the context of cancer therapy. A highly controversial and also emotionally discussed approach, the application of ascorbate in pharmacological doses, was first proposed and described by Pauling and Cameron in the 1970s (84, 85). Although their original hypothesis concerning the mode of action was incorrect (encapsulation of tumors by collagen induction), in the mean time it became evident that the anticancer effects of ascorbate are principally mediated by induction of radicals (1, 4). Furthermore, the crucial need for i.v. administration instead of oral supplementation to assure a sufficient, cytotoxic drug concentration has widely been acknowledged (86). This partly explains why the clinical observations of Pauling and Cameron could not be reproduced in clinical trials conducted in the 1980s. We recently observed functional effects of ascorbate on survival of melanoma cells (5). In the latter paper, we showed the cytotoxic effect of ascorbate on all 60 cancer cell lines of the NCI60 panel of cancer cells, which includes 9 human melanoma cell lines. The IC_50_ of ascorbate was 3.1 mM for all melanoma cell lines (0.2–8.5 mM); the overall IC_50_ of all 60 cancer cell lines was 4.5 mM. 8 mM ascorbate generated a high amount of intracellular peroxide radicals in LOX-IMVI melanoma cells leading to an increased percentage of sub-G1 (apoptotic) cells determined by FACS. In the melanoma cell lines, ascorbate treatment at the individual IC_50_ concentrations decreased GLUT-1 expression (pro-survival HIF-1α downstream target). In line, in the present paper, we observed a similar time-dependent prominent increase of BLM melanoma cells in the sub-G1 fraction, beginning at 12 h after incubation of the cells with 8 mM ascorbate.

Surprisingly, despite the abundance of scientific reports elucidating the mechanistic background of pharmacological ascorbate-induced cancer cell cytotoxicity, independent of the cellular mutation status, the successful transfer into the clinics has failed so far. The most likely explanations for this discrepancy are (i) the observation of induced ascorbate resistance by exogenous factors such as hypoxia present in metastatic tissue of cancer patients, which clinically has not been taken into consideration yet (5), and (ii) the existence of possible additional endogenous mechanistic features driven by ascorbate. Such additional effects might severely influence its cytotoxic efficacy for the treatment of cancer.

In the last few years, increasing evidence demonstrated that natural products and edibles harbor epigenetic activities, which might be beneficial for cancer therapy (24, 87). Epigenetic alterations that induce multiple changes in gene expression profiles are substantial features of cancer (88–90). Several epigenetic mechanisms have been described so far and the investigation of this complex molecular machinery is ongoing. Two principal mechanisms that cause a silencing of control genes and mediate tumor formation as well as tumor progression are the modulation of HDACs and the regulation of DNMTs. Therefore, in the present study, we investigated possible epigenetic impacts of ascorbate on melanoma cells to gain a more profound understanding of this alternative therapeutic approach widely used in complimentary medicine (91).

In particular, our *in silico* findings showed that ascorbate fits into the catalytic pocket of human HDACs and interacts with the zinc ion as well as other residues of the active site making it an interesting candidate that could act as a histone deacytelase inhibitor (HDACi). The obtained GoldScore values even assigned ascorbate to be a similar potent inhibitor as the well-known HDACi TSA. To verify these results, different HDAC inhibition assays were performed. However, neither the inhibition experiments with nuclear extracts nor the extensive profiling study could prove that ascorbate substantially inhibits classical human HDACs *in vitro*. Our second attempt was to test, if ascorbate can inhibit DNMTs within melanoma cells. Indeed, we could show that physiological levels of ascorbate in the micromolar range have no or a slightly activating activity on DNMTs, whereas pharmacological levels of ascorbate in the millimolar-range (achievable in patients via i.v.-administration) inhibit cellular DNMTs in melanoma cell lines. Based on these results, we conclude that ascorbate bears a novel DNMT inhibitory activity in high concentrations, but no HDAC-inhibitory potential.

Due to the newfound epigenetic activity of pharmacological ascorbate on DNMTs we next analyzed its impact on expression of miRNAs. The results of the chip analysis highlighted that upon stimulation of melanoma cells with either physiological or pharmacological ascorbate, a total of 151 miRNAs were differentially regulated in comparison to the untreated cells. Most interestingly, by comparing the melanoma cells incubated with the maximum physiological dose of 200 μM to cells pre-conditioned with pharmacological 8 mM to specifically analyze the impact of cytotoxicity-inducing drug concentrations, it became obvious that the majority of the up-regulated miRNAs are known to be involved in tumor suppression, cancer cell drug resistance, or inhibition of migration and invasion through inhibition of epithelial mesenchymal transition. The last-mentioned is a typical morphogenetic feature in the developing embryo untimely reappearing in cancer cells in general and in melanoma cells, in particular, due to their neural crest origin (92–94). Since the miRNAs found to be up-regulated upon ascorbate stimulation in the chip analysis were not validated by additional real-time PCR analyses, definite conclusions or a clear clinical significance cannot be drawn of the rather preliminary results yet. However, the up-regulated expression of miRNA due to ascorbate in melanoma cells correlated with an increased overall survival of breast cancer or nasopharyngeal carcinoma patients of the MIRUMIR database (28) with high expression of the respective miRNAs, therefore, suggesting a possible beneficial clinical relevance of the specific miRNA up-regulation by ascorbate. At this point, our results therefore allow us to generate the hypothesis that pharmacological ascorbate might modify the miRNA signature of melanoma cells, which subsequently might be beneficial for overall survival of melanoma patients in analogy to the endogenous miRNA expression profiles of breast cancer and nasopharyngeal carcinoma patients with either short- or long-term survival.

Considering the observed preliminary up-regulation of specific miRNAs by ascorbate (possibly via its novel DNMT inhibitory activity) governing a broad spectrum of tumor-suppressive effects including apoptosis induction, antiproliferative activity, and decrease of cancer cell invasion, this novel epigenetic signature of ascorbate might open the door for the exploration of ascorbate in combination with other classical or even epigenetically active molecules for cancer therapy (90) and therefore warrants further pre-clinical and clinical investigation.

## Author Contributions

Tobias W. Sinnberg, Alexander Berger, and Seema Noor: acquisition, analysis, and interpretation of data for the work; revising of the work critically for important intellectual content; final approval of the version to be published; agreement to be accountable for all aspects of the work. Mitchell Paul Levesque, Alexander Böcker, Heike Niessner, Ulrich M. Lauer, Michael Bitzer, and Claus Garbe: acquisition and analysis of data; revising of the work critically for important intellectual content; final approval of the version to be published; agreement to be accountable for all aspects of the work. Sascha Venturelli and Christian Busch: design of the work; analysis and interpretation of data; drafting of the manuscript; final approval of the version to be published; agreement to be accountable for all aspects of the work.

## Conflict of Interest Statement

The authors declare that the research was conducted in the absence of any commercial or financial relationships that could be construed as a potential conflict of interest.

## Footnotes

^1^http://www.ebi.ac.uk/pdbe/

^2^http://www.bioprofiling.de/MIRUMIR

## Abbreviations

DNMT, DNA methyltransferase; DNMTi, DNA methyltransferase inhibitor; HDAC, histone deacetylase; HDACi, histone deacetylase inhibitor; i.v., intravenous; miRNA, microRNA; ROS, reactive oxygen species; SAHA, suberoylanilide hydroxamic acid; TSA, trichostatin A.

## References

[1] Chen Q Espey MG Krishna MC Mitchell JB Corpe CP Buettner GR Pharmacologic ascorbic acid concentrations selectively kill cancer cells: action as a pro-drug to deliver hydrogen peroxide to tissues. Proc Natl Acad Sci U S A (2005) 102:13604–9 10.1073/pnas.0506390102 16157892 PMC1224653

[2] Chen Q Espey MG Sun AY Pooput C Kirk KL Krishna MC Pharmacologic doses of ascorbate act as a prooxidant and decrease growth of aggressive tumor xenografts in mice. Proc Natl Acad Sci U S A (2008) 105:11105–9 10.1073/pnas.0804226105 18678913 PMC2516281

[3] Du J Martin SM Levine M Wagner BA Buettner GR Wang SH Mechanisms of ascorbate-induced cytotoxicity in pancreatic cancer. Clin Cancer Res (2010) 16:509–20 10.1158/1078-0432.CCR-09-1713 20068072 PMC2807999

[4] Chen Q Espey MG Sun AY Lee JH Krishna MC Shacter E Ascorbate in pharmacologic concentrations selectively generates ascorbate radical and hydrogen peroxide in extracellular fluid in vivo. Proc Natl Acad Sci U S A (2007) 104:8749–54 10.1073/pnas.0702854104 17502596 PMC1885574

[5] Sinnberg T Noor S Venturelli S Berger A Schuler P Garbe C The ROS-induced cytotoxicity of ascorbate is attenuated by hypoxia and HIF-1alpha in the NCI60 cancer cell lines. J Cell Mol Med (2014) 18:530–41 10.1111/jcmm.12207 24330097 PMC3955158

[6] Kuiper C Dachs GU Currie MJ Vissers MC Intracellular ascorbate enhances hypoxia-inducible factor (HIF)-hydroxylase activity and preferentially suppresses the HIF-1 transcriptional response. Free Radic Biol Med (2014) 69:308–17 10.1016/j.freeradbiomed.2014.01.033 24495550

[7] Kuiper C Dachs GU Munn D Currie MJ Robinson BA Pearson JF Increased tumor ascorbate is associated with extended disease-free survival and decreased hypoxia-inducible factor-1 activation in human colorectal cancer. Front Oncol (2014) 4:10 10.3389/fonc.2014.00010 24551593 PMC3912592

[8] Kuiper C Molenaar IG Dachs GU Currie MJ Sykes PH Vissers MC Low ascorbate levels are associated with increased hypoxia-inducible factor-1 activity and an aggressive tumor phenotype in endometrial cancer. Cancer Res (2010) 70:5749–58 10.1158/0008-5472.CAN-10-0263 20570889

[9] Bram S Froussard P Guichard M Jasmin C Augery Y Sinoussi-Barre F Vitamin C preferential toxicity for malignant melanoma cells. Nature (1980) 284:629–31 10.1038/284629a0 7366735

[10] Meadows GG Pierson HF Abdallah RM Ascorbate in the treatment of experimental transplanted melanoma. Am J Clin Nutr (1991) 54:1284S–91S 1962584 10.1093/ajcn/54.6.1284s

[11] Kang JS Cho D Kim YI Hahm E Yang Y Kim D L-ascorbic acid (vitamin C) induces the apoptosis of B16 murine melanoma cells via a caspase-8-independent pathway. Cancer Immunol Immunother (2003) 52:693–8 10.1007/s00262-003-0407-6 12827307 PMC11032859

[12] Cha J Roomi MW Ivanov V Kalinovsky T Niedzwiecki A Rath M Ascorbate depletion increases growth and metastasis of melanoma cells in vitamin C deficient mice. Exp Oncol (2011) 33:226–30 22217712

[13] Cha J Roomi MW Ivanov V Kalinovsky T Niedzwiecki A Rath M Ascorbate supplementation inhibits growth and metastasis of B16FO melanoma and 4T1 breast cancer cells in vitamin C-deficient mice. Int J Oncol (2013) 42:55–64 10.3892/ijo.2012.1712 23175106 PMC3583641

[14] Schleich T Rodemeister S Venturelli S Sinnberg T Garbe C Busch C Decreased plasma ascorbate levels in stage IV melanoma patients. Metab Nutr Oncol (2013). 10.1055/s-0033-1348256

[15] Verrax J Calderon PB Pharmacologic concentrations of ascorbate are achieved by parenteral administration and exhibit antitumoral effects. Free Radic Biol Med (2009) 47:32–40 10.1016/j.freeradbiomed.2009.02.016 19254759

[16] Stephenson CM Levin RD Spector T Lis CG Phase I clinical trial to evaluate the safety, tolerability, and pharmacokinetics of high-dose intravenous ascorbic acid in patients with advanced cancer. Cancer Chemother Pharmacol (2013) 72:139–46 10.1007/s00280-013-2179-9 23670640 PMC3691494

[17] Hoffer LJ Levine M Assouline S Melnychuk D Padayatty SJ Rosadiuk K Phase I clinical trial of i.v. ascorbic acid in advanced malignancy. Ann Oncol (2008) 19:1969–74 10.1093/annonc/mdn377 18544557

[18] Welsh JL Wagner BA van’t Erve TJ Zehr PS Berg DJ Halfdanarson TR Pharmacological ascorbate with gemcitabine for the control of metastatic and node-positive pancreatic cancer (PACMAN): results from a phase I clinical trial. Cancer Chemother Pharmacol (2013) 71:765–75 10.1007/s00280-013-2070-8 23381814 PMC3587047

[19] Riordan HD Casciari JJ González MJ Riordan NH Miranda-Massari JR Taylor P A pilot clinical study of continuous intravenous ascorbate in terminal cancer patients. P R Health Sci J (2005) 24:269–76 16570523

[20] Ma Y Chapman J Levine M Polireddy K Drisko J Chen Q High-dose parenteral ascorbate enhanced chemosensitivity of ovarian cancer and reduced toxicity of chemotherapy. Sci Transl Med (2014) 6:222ra18 10.1126/scitranslmed.3007154 24500406

[21] Clarke JD Hsu A Yu Z Dashwood RH Ho E Differential effects of sulforaphane on histone deacetylases, cell cycle arrest and apoptosis in normal prostate cells versus hyperplastic and cancerous prostate cells. Mol Nutr Food Res (2011) 55:999–1009 10.1002/mnfr.201000547 21374800 PMC3129466

[22] Berger A Venturelli S Kallnischkies M Böcker A Busch C Weiland T Kaempferol, a new nutrition-derived pan-inhibitor of human histone deacetylases. J Nutr Biochem (2013) 24:977–85 10.1016/j.jnutbio.2012.07.001 23159065

[23] Venturelli S Berger A Böcker A Busch C Weiland T Noor S Resveratrol as a pan-HDAC inhibitor alters the acetylation status of histone proteins in human-derived hepatoblastoma cells. PLoS One (2013) 8:e73097 10.1371/journal.pone.0073097 24023672 PMC3758278

[24] Ellis L Atadja PW Johnstone RW Epigenetics in cancer: targeting chromatin modifications. Mol Cancer Ther (2009) 8:1409–20 10.1158/1535-7163.MCT-08-0860 19509247

[25] Chuang JC Jones PA Epigenetics and microRNAs. Pediatr Res (2007) 61:24R–9R 10.1203/pdr.0b013e3180457684 17413852

[26] Sato F Tsuchiya S Meltzer SJ Shimizu K microRNAs and epigenetics. FEBS J (2011) 278:1598–609 10.1111/j.1742-4658.2011.08089.x 21395977

[27] Lovat F Valeri N Croce CM microRNAs in the pathogenesis of cancer. Semin Oncol (2011) 38:724–33 10.1053/j.seminoncol.2011.08.006 22082758

[28] Antonov AV Knight RA Melino G Barlev NA Tsvetkov PO MIRUMIR: an online tool to test microRNAs as biomarkers to predict survival in cancer using multiple clinical data sets. Cell Death Differ (2013) 20:367 10.1038/cdd.2012.137 23175189 PMC3554342

[29] Carey TE Takahashi T Resnick LA Oettgen HF Old LJ Cell surface antigens of human malignant melanoma: mixed hemadsorption assays for humoral immunity to cultured autologous melanoma cells. Proc Natl Acad Sci U S A (1976) 73:3278–82 1067619 10.1073/pnas.73.9.3278 PMC431008

[30] Lockshin A Giovanella BC De Ipolyi PD Williams LJ Jr Mendoza JT Yim SO Exceptional lethality for nude mice of cells derived from a primary human melanoma. Cancer Res (1985) 45:345–50 3965144

[31] Labute P Protonate3D: assignment of ionization states and hydrogen coordinates to macromolecular structures. Proteins (2009) 75:187–205 10.1002/prot.22234 18814299 PMC3056144

[32] Reischauer S Lgl2 executes its function as a tumor suppressor by regulating ErbB signaling in the zebrafish epidermis. PLoS Genet (2009) 5:e1000720 10.1371/journal.pgen.1000720 19911055 PMC2771016

[33] Lim SO Gu JM Kim MS Kim HS Park YN Park CK Epigenetic changes induced by reactive oxygen species in hepatocellular carcinoma: methylation of the E-cadherin promoter. Gastroenterology (2008) 135:2128–40 10.1053/j.gastro.2008.07.027 18801366

[34] Williams SV Platt FM Hurst CD Aveyard JS Taylor CF Pole JC High-resolution analysis of genomic alteration on chromosome arm 8p in urothelial carcinoma. Genes Chromosomes Cancer (2010) 49(7):642–59 10.1002/gcc.20775 20461757

[35] Costa FF Bischof JM Vanin EF Lulla RR Wang M Sredni ST Identification of microRNAs as potential prognostic markers in ependymoma. PLoS One (2011) 6(10):e25114 10.1371/journal.pone.0025114 22053178 PMC3203863

[36] Anwar SL Albat C Krech T Hasemeier B Schipper E Schweitzer N Concordant hypermethylation of intergenic microRNA genes in human hepatocellular carcinoma as new diagnostic and prognostic marker. Int J Cancer (2013) 133(3):660–70 10.1002/ijc.28068 23364900

[37] Endo H Muramatsu T Furuta M Uzawa N Pimkhaokham A Amagasa T Potential of tumor-suppressive miR-596 targeting LGALS3BP as a therapeutic agent in oral cancer. Carcinogenesis (2013) 34(3):560–9 10.1093/carcin/bgs376 23233740

[38] Galluzzi L Morselli E Vitale I Kepp O Senovilla L Criollo A miR-181a and miR-630 regulate cisplatin-induced cancer cell death. Cancer Res (2010) 70(5):1793–803 10.1158/0008-5472.CAN-09-3112 20145152

[39] Kuo TC Tan CT Chang YW Hong CC Lee WJ Chen MW Angiopoietin-like protein 1 suppresses SLUG to inhibit cancer cell motility. J Clin Invest (2013) 123(3):1082–95 10.1172/JCI64044 23434592 PMC3582121

[40] Farhana L Dawson MI Murshed F Das JK Rishi AK Fontana JA Upregulation of miR-150* and miR-630 induces apoptosis in pancreatic cancer cells by targeting IGF-1R. PLoS One (2013) 8(5):e61015 10.1371/journal.pone.0061015 23675407 PMC3651232

[41] Gougelet A Pissaloux D Besse A Perez J Duc A Dutour A miRNA profiles in osteosarcoma as a predictive tool for ifosfamide response. Int J Cancer (2011) 129(3):680–90 10.1002/ijc.25715 20949564

[42] Han Y Chen J Zhao X Liang C Wang Y Sun L microRNA expression signatures of bladder cancer revealed by deep sequencing. PLoS One (2011) 6(3):e18286 10.1371/journal.pone.0018286 21464941 PMC3065473

[43] Bhatti I Lee A James V Hall RI Lund JN Tufarelli C Knockdown of microRNA-21 inhibits proliferation and increases cell death by targeting programmed cell death 4 (PDCD4) in pancreatic ductal adenocarcinoma. J Gastrointest Surg (2011) 15(1):199–208 10.1007/s11605-010-1381-x 21088996

[44] Ding L Xu Y Zhang W Deng Y Si M Du Y MiR-375 frequently downregulated in gastric cancer inhibits cell proliferation by targeting JAK2. Cell Res (2010) 20(7):784–93 10.1038/cr.2010.79 20548334

[45] Tsukamoto Y Nakada C Noguchi T Tanigawa M Nguyen LT Uchida T microRNA-375 is downregulated in gastric carcinomas and regulates cell survival by targeting PDK1 and 14-3-3zeta. Cancer Res (2010) 70(6):2339–49 10.1158/0008-5472.CAN-09-2777 20215506

[46] Liu AM Poon RT Luk JM microRNA-375 targets hippo-signaling effector YAP in liver cancer and inhibits tumor properties. Biochem Biophys Res Commun (2010) 394(3):623–7 10.1016/j.bbrc.2010.03.036 20226166

[47] Hui AB Lenarduzzi M Krushel T Waldron L Pintilie M Shi W Comprehensive microRNA profiling for head and neck squamous cell carcinomas. Clin Cancer Res (2010) 16(4):1129–39 10.1158/1078-0432.CCR-09-2166 20145181

[48] Mathé EA Nguyen GH Bowman ED Zhao Y Budhu A Schetter AJ microRNA expression in squamous cell carcinoma and adenocarcinoma of the esophagus: associations with survival. Clin Cancer Res (2009) 15(19):6192–200 10.1158/1078-0432.CCR-09-1467 19789312 PMC2933109

[49] Bierkens M Krijgsman O Wilting SM Bosch L Jaspers A Meijer GA Focal aberrations indicate EYA2 and hsa-miR-375 as oncogene and tumor suppressor in cervical carcinogenesis. Genes Chromosomes Cancer (2013) 52(1):56–68 10.1002/gcc.22006 22987659

[50] Wilting SM Verlaat W Jaspers A Makazaji NA Agami R Meijer CJ Methylation-mediated transcriptional repression of microRNAs during cervical carcinogenesis. Epigenetics (2013) 8(2):220–8 10.4161/epi.23605 23324622 PMC3592908

[51] Necela BM Carr JM Asmann YW Thompson EA Differential expression of microRNAs in tumors from chronically inflamed or genetic (APC) models of colon cancer. PLoS One (2011) 6(4):e18501 10.1371/journal.pone.0018501 21532750 PMC3075242

[52] Saini S Yamamura S Majid S Shahryari V Hirata H Tanaka Y microRNA-708 induces apoptosis and suppresses tumorigenicity in renal cancer cells. Cancer Res (2011) 71(19):6208–19 10.1158/0008-5472.CAN-11-0073 21852381 PMC3940359

[53] Saini S Majid S Shahryari V Arora S Yamamura S Chang I miRNA-708 control of CD44(+) prostate cancer-initiating cells. Cancer Res (2012) 72(14):3618–30 10.1158/0008-5472.CAN-12-0540 22552290

[54] Guo P Lan J Ge J Nie Q Mao Q Qiu Y miR-708 acts as a tumor suppressor in human glioblastoma cells. Oncol Rep (2013) 30(2):870–6 10.3892/or.2013.2526 23754151

[55] Östling P Leivonen SK Aakula A Kohonen P Mäkelä R Hagman Z Systematic analysis of microRNAs targeting the androgen receptor in prostate cancer cells. Cancer Res (2011) 71(5):1956–67 10.1158/0008-5472.CAN-10-2421 21343391

[56] Navon R Wang H Steinfeld I Tsalenko A Ben-Dor A Yakhini Z Novel rank-based statistical methods reveal microRNAs with differential expression in multiple cancer types. PLoS One (2009) 4(11):e8003 10.1371/journal.pone.0008003 19946373 PMC2777376

[57] Hatziapostolou M Polytarchou C Aggelidou E Drakaki A Poultsides GA Jaeger SA An HNF4α-miRNA inflammatory feedback circuit regulates hepatocellular oncogenesis. Cell (2011) 147(6):1233–47 10.1016/j.cell.2011.10.043 22153071 PMC3251960

[58] Rokah OH Granot G Ovcharenko A Modai S Pasmanik-Chor M Toren A Downregulation of miR-31, miR-155, and miR-564 in chronic myeloid leukemia cells. PLoS One (2012) 7(4):e35501 10.1371/journal.pone.0035501 22511990 PMC3325224

[59] Pogribny IP Filkowski JN Tryndyak VP Golubov A Shpyleva SI Kovalchuk O Alterations of microRNAs and their targets are associated with acquired resistance of MCF-7 breast cancer cells to cisplatin. Int J Cancer (2010) 127(8):1785–94 10.1002/ijc.25191 20099276

[60] Tang JT Wang JL Du W Hong J Zhao SL Wang YC microRNA 345, a methylation-sensitive microRNA is involved in cell proliferation and invasion in human colorectal cancer. Carcinogenesis (2011) 32(8):1207–15 10.1093/carcin/bgr114 21665895

[61] Yamada H Yanagisawa K Tokumaru S Taguchi A Nimura Y Osada H Detailed characterization of a homozygously deleted region corresponding to a candidate tumor suppressor locus at 21q11-21 in human lung cancer. Genes Chromosomes Cancer (2008) 47(9):810–8 10.1002/gcc.20582 18523997

[62] White NM Chow TF Mejia-Guerrero S Diamandis M Rofael Y Faragalla H Three dysregulated miRNAs control kallikrein 10 expression and cell proliferation in ovarian cancer. Br J Cancer (2010) 102(8):1244–53 10.1038/sj.bjc.6605634 20354523 PMC2856011

[63] Ju X Li D Shi Q Hou H Sun N Shen B Differential microRNA expression in childhood B-cell precursor acute lymphoblastic leukemia. Pediatr Hematol Oncol (2009) 26(1):1–10 10.1080/08880010802378338 19206004

[64] Holleman A Chung I Olsen RR Kwak B Mizokami A Saijo N miR-135a contributes to paclitaxel resistance in tumor cells both in vitro and in vivo. Oncogene (2011) 30(43):4386–98 10.1038/onc.2011.148 21552288 PMC3572709

[65] Zhou L Qiu T Xu J Wang T Wang J Zhou X miR-135a/b modulate cisplatin resistance of human lung cancer cell line by targeting MCL1. Pathol Oncol Res (2013) 19(4):677–83 10.1007/s12253-013-9630-4 23640248

[66] Jukic DM Rao UN Kelly L Skaf JS Drogowski LM Kirkwood JM microRNA profiling analysis of differences between the melanoma of young adults and older adults. J Transl Med (2010) 8:27 10.1186/1479-5876-8-27 20302635 PMC2855523

[67] Liu SG Qin XG Zhao BS Qi B Yao WJ Wang TY Differential expression of miRNAs in esophageal cancer tissue. Oncol Lett (2013) 5(5):1639–42 23761828 10.3892/ol.2013.1251 PMC3678876

[68] Waltering KK Porkka KP Jalava SE Urbanucci A Kohonen PJ Latonen LM Androgen regulation of micro-RNAs in prostate cancer. Prostate (2011) 71(6):604–14 10.1002/pros.21276 20945501

[69] Guled M Lahti L Lindholm PM Salmenkivi K Bagwan I Nicholson AG CDKN2A, NF2, and JUN are dysregulated among other genes by miRNAs in malignant mesothelioma-A miRNA microarray analysis. Genes Chromosomes Cancer (2009) 48(7):615–23 10.1002/gcc.20669 19396864

[70] Jia L Wu J Zhang L Chen J Zhong D Xu S Restoration of miR-1228* expression suppresses epithelial-mesenchymal transition in gastric cancer. PLoS One (2013) 8(3):e58637 10.1371/journal.pone.0058637 23554909 PMC3595239

[71] Li Y Mine T Ioannides CG Short GC-rich RNA similar to miR 1909 and 1915 folds in silico with the 5’-UTR and ORF of Notch and responders: potential for the elimination of cancer stem cells. Oncol Rep (2010) 24(6):1443–53 10.3892/or_00001004 21042738

[72] Xu K Liang X Cui D Wu Y Shi W Liu J miR-1915 inhibits Bcl-2 to modulate multidrug resistance by increasing drug-sensitivity in human colorectal carcinoma cells. Mol Carcinog (2013) 52(1):70–8 10.1002/mc.21832 22121083

[73] Guo L Liu Y Bai Y Sun Y Xiao F Guo Y Gene expression profiling of drug-resistant small cell lung cancer cells by combining microRNA and cDNA expression analysis. Eur J Cancer (2010) 46(9):1692–702 10.1016/j.ejca.2010.02.043 20371173

[74] Li J Wang Y Luo J Fu Z Ying J Yu Y miR-134 inhibits epithelial to mesenchymal transition by targeting FOXM1 in non-small cell lung cancer cells. FEBS Lett (2012) 586(20):3761–5 10.1016/j.febslet.2012.09.016 23010597

[75] Niu CS Yang Y Cheng CD MiR-134 regulates the proliferation and invasion of glioblastoma cells by reducing nanog expression. Int J Oncol (2013) 42(5):1533–40 10.3892/ijo.2013.1844 23467648 PMC3661226

[76] Yin C Wang PQ Xu WP Yang Y Zhang Q Ning BF Hepatocyte nuclear factor-4α reverses malignancy of hepatocellular carcinoma through regulating miR-134 in the DLK1-DIO3 region. Hepatology (2013) 58(6):1964–76 10.1002/hep.26573 23775631

[77] Pigati L Yaddanapudi SC Iyengar R Kim DJ Hearn SA Danforth D Selective release of microRNA species from normal and malignant mammary epithelial cells. PLoS One (2010) 5(10):e13515 10.1371/journal.pone.0013515 20976003 PMC2958125

[78] Tili E Michaille JJ Alder H Volinia S Delmas D Latruffe N Resveratrol modulates the levels of microRNAs targeting genes encoding tumor-suppressors and effectors of TGFβ signaling pathway in SW480 cells. Biochem Pharmacol (2010) 80(12):2057–65 10.1016/j.bcp.2010.07.003 20637737 PMC3918904

[79] Pan J Hu H Zhou Z Sun L Peng L Yu L Tumor-suppressive mir-663 gene induces mitotic catastrophe growth arrest in human gastric cancer cells. Oncol Rep (2010) 24(1):105–12 10.3892/or_00000834 20514450

[80] Sand M Skrygan M Sand D Georgas D Gambichler T Hahn SA Comparative microarray analysis of microRNA expression profiles in primary cutaneous malignant melanoma, cutaneous malignant melanoma metastases, and benign melanocytic nevi. Cell Tissue Res (2013) 351(1):85–98 10.1007/s00441-012-1514-5 23111773

[81] Eigentler TK Figl A Krex D Mohr P Mauch C Rass K Number of metastases, serum lactate dehydrogenase level, and type of treatment are prognostic factors in patients with brain metastases of malignant melanoma. Cancer (2011) 117:1697–703 10.1002/cncr.25631 21472716

[82] Garbe C Peris K Hauschild A Saiag P Middleton M Spatz A Diagnosis and treatment of melanoma. European consensus-based interdisciplinary guideline – update 2012. Eur J Cancer (2012) 48:2375–90 10.1016/j.ejca.2012.06.013 22981501

[83] Garbe C Abusaif S Eigentler TK Vemurafenib. Recent Results Cancer Res (2014) 201:215–25 10.1007/978-3-642-54490-3_13 24756795

[84] Cameron E Pauling L Supplemental ascorbate in the supportive treatment of cancer: prolongation of survival times in terminal human cancer. Proc Natl Acad Sci U S A (1976) 73:3685–9 10.1073/pnas.73.10.3685 1068480 PMC431183

[85] Cameron E Pauling L Supplemental ascorbate in the supportive treatment of cancer: reevaluation of prolongation of survival times in terminal human cancer. Proc Natl Acad Sci U S A (1978) 75:4538–42 10.1073/pnas.75.9.4538 279931 PMC336151

[86] Mikirova N Casciari J Riordan N Hunninghake R Clinical experience with intravenous administration of ascorbic acid: achievable levels in blood for different states of inflammation and disease in cancer patients. J Transl Med (2013) 11:191 10.1186/1479-5876-11-191 23947403 PMC3751545

[87] Meeran SM Ahmed A Tollefsbol TO Epigenetic targets of bioactive dietary components for cancer prevention and therapy. Clin Epigenetics (2010) 1:101–16 10.1007/s13148-010-0011-5 21258631 PMC3024548

[88] Venturelli S Berger A Weiland T Zimmermann M Häcker S Peter C Dual antitumour effect of 5-azacytidine by inducing a breakdown of resistance-mediating factors and epigenetic modulation. Gut (2011) 60:156–65 10.1136/gut.2010.208041 21106551

[89] Weiland T Berger A Essmann F Lauer UM Bitzer M Venturelli S Kinetic tracking of therapy-induced senescence using the real-time cell analyzer single plate system. Assay Drug Dev Technol (2012) 10:289–95 10.1089/adt.2011.0402 22192307

[90] Venturelli S Berger A Weiland T Essmann F Waibel M Nuebling T Differential induction of apoptosis and senescence by the DNA methyltransferase inhibitors 5-azacytidine and 5-aza-2’-deoxycytidine in solid tumor cells. Mol Cancer Ther (2013) 12:2226–36 10.1158/1535-7163.MCT-13-0137 23924947

[91] Padayatty SJ Sun AY Chen Q Espey MG Drisko J Levine M Vitamin C: intravenous use by complementary and alternative medicine practitioners and adverse effects. PLoS One (2010) 5:e11414 10.1371/journal.pone.0011414 20628650 PMC2898816

[92] Busch C Krochmann J Drews U The chick embryo as an experimental system for melanoma cell invasion. PLoS One (2013) 8:e53970 10.1371/journal.pone.0053970 23342051 PMC3544663

[93] Schriek G Oppitz M Busch C Just L Drews U Human SK-Mel 28 melanoma cells resume neural crest cell migration after transplantation into the chick embryo. Melanoma Res (2005) 15:225–34 10.1097/00008390-200508000-00001 16034299

[94] Busch C Drews U Garbe C Eisele SR Oppitz M Neural crest cell migration of mouse B16-F1 melanoma cells transplanted into the chick embryo is inhibited by the BMP-antagonist noggin. Int J Oncol (2007) 31:1367–78 10.3892/ijo.31.6.1367 17982664

